# On Sahlqvist Formulas in Relevant Logic

**DOI:** 10.1007/s10992-017-9445-y

**Published:** 2017-08-22

**Authors:** Guillermo Badia

**Affiliations:** Department of Knowledge-Based Mathematical Systems, Johannes Kepler Universität, Linz, Austria

**Keywords:** Relevant logic, Routley-Meyer semantics, Correspondence theory, Frame definability, Sahlqvist’s correspondence

## Abstract

This paper defines a Sahlqvist fragment for relevant logic and establishes that each class of frames in the Routley-Meyer semantics which is definable by a Sahlqvist formula is also elementary, that is, it coincides with the class of structures satisfying a given first order property calculable by a Sahlqvist-van Benthem algorithm. Furthermore, we show that some classes of Routley-Meyer frames definable by a relevant formula are not elementary.

## Introduction

The *Sahlqvist Correspondence Theorem* is a celebrated result in modal logic (see [3, 6] for slightly different expositions). It tells us that when a modal formula *ϕ* has certain syntactic form, we can always compute a first order formula *ψ* *in the signature of Kripke frames* such that a frame validates *ϕ* iff it satisfies condition *ψ*, so the class of frames definable by *ϕ* is elementary. The proof relies on the so called “Sahlqvist-van Benthem algorithm” for transforming second order frame correspondents of some modal formulas into first order properties.

The present note is a contribution to the correspondence theory of relevant logic in the Routley-Meyer semantic framework (cf. [4, 8, 16]).^1^ The framework has been considerably generalized and investigated in relation to other semantics in [1].

We will show that, *mutatis mutandis*, the argument for the Sahlqvist Correspondence Theorem (as presented in [3]) can be adapted to the context of relevant logic to prove an analogous result in the Routley-Meyer semantics. This result improves our understanding of the first order properties of Routley-Meyer frames which are definable in relevant logic. It also begins to solve Problem 8.4.18 from [1]. Moreover, we will show that some relevant formulas actually define non-elementary properties of frames, so the problem of elementarity is not trivial.

Correspondence theory for the broader setting of substructural logics with frame semantics was briefly explored by Restall in [14] (pp. 263-265) although the subject appears to have been discussed for the first time in the relevant logic literature in [13].^2^ Recently, Suzuki [18] provided a rather general Sahlqvist result for substructural logics with respect to what he calls *bi-approximation semantics* (which unfortunately is much more complicated than the Routley-Meyer framework). Other settings without boolean negation where Sahlqvist theorems have been obtained are positive modal logic [5] and relevant modal logic [17]. In particular, the work in [17] is rather close to ours, but the concern there is still modal logic.

In Section 2, we will introduce Routley-Meyer frames and some basic propositions on frame correspondence. In Section 3, we will discuss elementary classes of Routley-Meyer frames and prove that not all classes of Routley-Meyer frames definable by a relevant formula are elementary. In Section 4, we will present a Sahlqvist fragment of relevant languages and establish a Sahlqvist correspondence theorem for the Routley-Meyer semantics. Finally, in Section 5, we will sum up our work.

## Routley-Meyer Frames

In this paper, a *relevant language* $\mathcal {L}$ will contain a countable list PROP of propositional variables *p*, *q*, *r*… and the logical symbols: ∼ (negation), ∧ (conjunction), ∨ (disjunction), ∘ (fusion), → (implication), **t** (the Ackermann constant). Formulas are constructed in the usual way:

$$\phi ::= p \ | \ \textbf{t} \ | \ \sim \phi \ | \ \phi \wedge \psi \ | \ \phi \vee \psi \ |\ \phi \rightarrow \psi \ | \ \phi \circ \psi. $$

In this paper, a Routley-Meyer *frame* for $\mathcal {L}$ is a structure $\mathfrak {F} = \langle W, R, *, O \rangle $, where *W* is a non-empty set, *∅*≠*O* ⊆ *W*, ∗ is an operation ∗ : *W*→*W*, and *R* ⊆ *W* × *W* × *W* satisfies p1-p5 below. In the standard way, we will abbreviate ∃*z*(*O* *z* ∧ *R* *z* *x* *y*) by *x* ≤ *y*.

- p1. *x* ≤ *x*
- p2. If *x* ≤ *y* and *R* *y* *z* *v* then *R* *x* *z* *v*.
- p3. If *x* ≤ *y* and *R* *z* *y* *v* then *R* *z* *x* *v*.
- p4. If *x* ≤ *y* and *R* *z* *v* *x* then *R* *z* *v* *y*.
- p5. If *x* ≤ *y* then *y* ^∗^≤ *x* ^∗^ .
- p6. *O* is upward closed under ≤.

The relation ≤ is a preorder. We can see this as follows. By p1, we have reflexivity. Now if *x* ≤ *y* (i.e., ∃*u*(*O* *u* ∧ *R* *u* *x* *y*)) and *y* ≤ *z* (i.e., ∃*v*(*O* *v* ∧ *R* *v* *y* *z*)), by p3, we have that ∃*v*(*O* *v* ∧ *R* *v* *x* *z*), i.e., *x* ≤ *z*.

These frames are essentially the same as what are called **B** ^∘**t**^-frames in [4] (p. 80) except that we have left out the condition that ∗ is an involution. Hence, our starting basic system of relevant logic is slightly weaker than **B** ^∘**t**^ from [4]. The reason for this is that we are interested in finding the exact first order frame correspondents of the double negation laws ∼∼ *p* → *p* and *p* →∼∼ *p* using our Sahlqvist-van Benthem algorithm, but this task becomes trivial if ∗ is an involution. Indeed, the system is just the Hilbert calculus **B** ^∘**t**^ presented in [4] (p. 74) minus the axiom ∼∼ *p* → *p*, whose axioms and rules we review here for the sake of completeness (a substitution rule will be tacitly assumed):

- A1. **t**
- A2. **t** → (*p* → *p*)
- A3. *p* ∧ *q* → *p*
- A4. *p* ∧ *q* → *q*
- A5. (*p* → *q*) ∧ (*p* → *r*) → (*p* → *q* ∧ *r*)
- A6. *p* → *p* ∨ *q*
- A7. *q* → *p* ∨ *q*
- A8. *p* ∧ (*q* ∨ *r*) → (*p* ∧ *q*) ∨ (*p* ∧ *r*)
- R1. *p*,*p* → *q*/*q*
- R2. *p*,*q*/*p* ∧ *q*
- R3. *p* → *q*/(*q* → *r*) → (*p* → *r*)
- R4. *p* → *q*/(*r* → *p*) → (*r* → *q*)
- R5. *p* →∼ *q*/*q* →∼ *p*
- R6. (*p* ∘ *q*) → *r*/*p* → (*q* → *r*)
- R7. *p* → (*q* → *r*)/(*p* ∘ *q*) → *r*

A Routley-Meyer *model* for $\mathcal {L}$ is a pair $\langle \mathfrak {F}, V\rangle $, where *V* : PROP → ℘ (*W*) is a function such that for any *p* ∈ PROP, *V* (*p*) is upward closed under the ≤ relation, that is, *x* ∈ *V* (*p*) and *x* ≤ *y* implies that *y* ∈ *V* (*p*). We define satisfaction at *w* in *M* recursively as follows:

*M, w*$\Vdash $**t**
iff there is *v* ∈ *O* ^*M*^ such that *v* ≤ *w*
*M, w*$\Vdash $ p
iff *w* ∈ *V* (*p*),
*M, w*$\Vdash \sim \phi $
iff $M, w^{*} \nVdash \phi $,
*M, w*$\Vdash \phi \wedge \psi $
iff $M, w \Vdash \phi $ and $M, w \Vdash \psi $,
*M, w*$\Vdash \phi \vee \psi $
iff $M, w \Vdash \phi $ or $M, w \Vdash \psi $,
*M, w*$\Vdash \phi \rightarrow \psi $
iff for every *a, b* such that *R* ^*M*^ *w* *a* *b*, if $M, a \Vdash \phi $ then *M, b* $\Vdash \psi $,
*M, w*$\Vdash \phi \circ \psi $
iff there are *a, b* such that *R* ^*M*^ *a* *b* *w*, $M, a \!\Vdash \! \phi $ and $M, b \Vdash \psi $.

A formula *ϕ* is said to be *true* in a model *M* if $M, w \Vdash \phi $ for all *w* ∈ *O*. *ϕ* is said to be *valid* in a frame $\mathfrak {F}$ (in symbols $\mathfrak {F} \Vdash \phi $) if *ϕ* is true in any model *M* based on $\mathfrak {F}$. When *w* is an arbitrary world of $\mathfrak {F}$, we also write $\mathfrak {F}, w \Vdash \phi $ to mean that for every model *M* based on $\mathfrak {F}$, $M, w \Vdash \phi $. Validity and this latter semantic notions will be the focus of the present study. We will be interested in what the languages defined above can say at the level of frames, not so much of models.

### **Lemma 1**

(Hereditary Lemma) *For any relevant formula* *ϕ* *,* *model* M *based on a Routley-Meyer frame, and worlds* *x*,*y* *of* M*,* *x* ≤ *y* *implies that* $M, x \Vdash \phi $ *only if* $M, y \Vdash \phi $ *.*

### *Proof*

This is traditionally proven by induction on formula complexity (see [8]). The case *ϕ* = *p* follows simply because *V* (*p*) is upward closed under ≤, while the case *ϕ* = **t** follows because ≤ is a transitive relation.

Next let *ϕ* =∼ *ψ*. By p5, we must have that *y* ^∗^≤ *x* ^∗^ given our assumption that *x* ≤ *y*. Now, if $M, x \Vdash \sim \psi $ then $M, x^{*} \nVdash \psi $, and by inductive hypothesis, it follows that $M, y^{*} \nVdash \psi $, so $M, y \Vdash \sim \psi $ as desired.

Let *ϕ* = *ψ* → *χ*. If $M, x \Vdash \psi \rightarrow \chi $, then for every *a*,*b* such that *R* *x* *a* *b*, if $M, a \Vdash \psi $ then $M, b \Vdash \psi $. Now suppose that $M, y \nVdash \psi \rightarrow \chi $, so there are *a* ^′^,*b* ^′^ such that *R* *y* *a* ^′^ *b* ^′^ while $M, a^{\prime } \Vdash \psi $ and $M, b^{\prime } \nVdash \chi $. However, by p2, it must be that *R* *x* *a* ^′^ *b* ^′^, which is impossible given the assumption that $M, x \Vdash \psi \rightarrow \chi $. Hence, actually $M, y \Vdash \psi \rightarrow \chi $.

Let *ϕ* = *ψ* ∘ *χ*. Similar to the previous case but appealing to p4 instead. The remaining cases are trivial. □

Consider a monadic second order language that comes with one function symbol ∗, a unary predicate *O*, a distinguished three place relation symbol *R*, and a unary predicate variable *P* for each *p* ∈ PROP. Following the tradition in modal logic, we might call this a *correspondence language* $\mathcal {L}^{corr}$ for $\mathcal {L}$ (cf. [3]). Now we can read a model *M* as a model for $\mathcal {L}^{corr}$ in a straightforward way: *W* is taken as the domain of the structure, *V* specifies the denotation of each of the predicates *P*,*Q*,…, the collection *O* is the object assigned to the predicate *O*, while ∗ is the denotation of the function symbol ∗ of $\mathcal {L}^{corr}$ and *R* the denotation of the relation *R* of $\mathcal {L}^{corr}$.

Where *t* is a term in the correspondence language, we write *ϕ* ^*t*/*x*^ for the result of replacing *x* with *t* everywhere in the formula *ϕ*. As expected, it is easy to specify a translation from the formulas of the relevant language into formulas of first order logic with *one free variable* as follows:

$$\begin{array}{@{}rcl@{}} T_{x}(\textbf{t}) &=& Ox\\ T_{x}(p) &=& Px\\ T_{x}(\sim \phi) &=& \neg T_{x}(\phi)^{x^{*}/x}\\ T_{x}(\phi \wedge \psi) &=& T_{x}(\phi) \wedge T_{x}(\psi)\\ T_{x}(\phi \vee \psi) &=& T_{x}(\phi) \vee T_{x}(\psi)\\ T_{x}(\phi \rightarrow \psi) &=& \forall y, z (Rxyz \wedge T_{x}(\phi)^{y/x} \supset T_{x}(\psi)^{z/x})\\ T_{x}(\phi \circ \psi) &=& \exists y, z (Ryzx \wedge T_{x}(\phi)^{y/x} \wedge T_{x}(\psi)^{z/x}). \end{array} $$

The symbols ¬ and ⊃ represent boolean negation and material implication in classical logic (which should not be confused with the relevant ∼ and →).

Next we prove a proposition to the effect that our proposed translation is adequate. While $\Vdash $ stands for satisfaction as defined for relevant languages, $\vDash $ will be the usual Tarskian satisfaction relation from classical logic.

### **Proposition 2**

*For any* w*,* $M, w \Vdash \phi $ *if and only if* $M \vDash T_{x}(\phi ) [w]$ *.*

Hence, without loss of generality, we can switch (when convenient) to the following presentation of $\mathcal {L}$ as a fragment of the classical language $\mathcal {L}^{corr}$:

- $\phi ::= Px \ | \ \exists v (Ov \wedge v \leq x) \ | \ \neg \phi ^{x^{*}/x} \ | \ \phi \wedge \psi \ | \ \phi \vee \psi \ |\ \forall y, z (Rxyz \wedge \phi ^{y/x} \supset \psi ^{z/x}) \ | \ \exists y, z (Ryzx \wedge \phi ^{y/x} \wedge \psi ^{z/x})$.

Working with this presentation makes life easier in the next definition.

### **Definition 1**

A relevant formula *ϕ* is said to be positive if *T*_*x*_(*ϕ*) is a formula built up from atomic formulas involving only unary predicates and first order formulas where the only non-logical symbols are R and O, using the connectives ∃,∀,∧ and ∨. On the other hand *ϕ* is negative if *T*_*x*_(*ϕ*) is a formula built up from boolean negations of atomic formulas involving only unary predicates and first order formulas where the only non-logical symbols are R and T, using the connectives ∃,∀,∧ and ∨.

A remark seems in order here: the translation of a formula of the form *ϕ* → *ψ* is in general not positive as it involves ⊃ with its usual definition in terms of ¬ and ∨.

### **Lemma 3**

*Let* M *be a Routley-Meyer model for a relevant language* $\mathcal {L}$ *,* $\phi (\overline {x})$ *a positive first order formula of* $\mathcal {L}^{corr}$ *where* $\overline {x}$ *is a sequence of variables, and* *M* ^′^ *a Routley-Meyer model identical to* M *except that the interpretation* *of the unary predicates of the signature of* M *in the model* *M* ^′^ *are supersets of the interpretation of the corresponding* *predicates in* M*. Then for any sequence of elements* $\overline {a}$ *of* M*,* $M \vDash \phi [\overline {a}]$ *only if* $M^{\prime } \vDash \phi [\overline {a}]$ *.*

### *Proof*

This follows from Theorem 10.3.3 (a) in [11]. □

We say that a formula *ϕ* ^′^ of $\mathcal {L}^{corr}$ *corresponds* to (or is a *correspondent* of) a relevant formula *ϕ* if for any Routley-Meyer frame $\mathfrak {F}$, $\mathfrak {F} \Vdash \phi $ iff $\mathfrak {F} \vDash \phi ^{\prime }$. A formula *ψ* of $\mathcal {L}^{corr}$ *locally corresponds* to a relevant formula *ϕ* if for any Routley-Meyer frame $\mathfrak {F}$ and world *w* of $\mathfrak {F}$, $\mathfrak {F}, w \Vdash \phi $ iff $\mathfrak {F} \vDash \psi [w]$.

If we abreviate the formula of $\mathcal {L}^{corr}$ which expresses that the value of a given predicate *P* is upward closed under ≤ by *U* *p*_≤_(*P*), the next proposition is easily established using Proposition 2.

### **Proposition 4**

*For any relevant formula* *ϕ*(*p*_1_,…, *p*_*n*_)*,* *Routley-Meyer frame* $\mathfrak {F}$ *and world* w *of* $\mathfrak {F}$ *,* *the following holds:*

- *(i)* $\mathfrak {F} \Vdash \phi $ *iff* $\mathfrak {F} \vDash \forall P_{1}, \dots , P_{n} (Up_{\leq }(P_{1}) \wedge {\dots } \wedge Up_{\leq }(P_{n}) \supset \forall w (Ow \supset T_{x}(\phi )^{w/x}))$
- *(ii)* $\mathfrak {F}, w \Vdash \phi $ *iff* $\mathfrak {F} \vDash \forall P_{1}, \dots , P_{n} (Up_{\leq }(P_{1}) \wedge {\dots } \wedge Up_{\leq }(P_{n}) \supset T_{x}(\phi ))[w]$ *.*

A class (or, informally, a property) *K* of Routley-Meyer frames will be said to be *definable* by a relevant formula *ϕ* if *K* is exactly the class of all frames validating *ϕ*. So, at the level of frames and validity, relevant languages can be seen as fragments of monadic second order logic as opposed to fragments of first order logic at the level of models. To be precise, the “relevant fragment” of monadic second order logic can be taken to be any formula equivalent to a formula in the set

{∀*P*_1_,…,*P*_*n*_(*U* *p*_≤_(*P*_1_) ∧… ∧ *U* *p*_≤_(*P*_*n*_) ⊃ *T*_*x*_(*ϕ*)) : *ϕ* is a relevant formula}.

## Elementary Classes of Frames

A class of structures *K* is said to be *elementary* if there is a first order formula *ϕ* such that *K* is identical to the class of all models of *ϕ*. Since frames are clearly structures, then it makes sense to talk about elementary classes of frames.

Note that it is easy to prove that a class of Routley-Meyer frames which is definable by a formula of relevant logic is elementary iff it is closed under ultraproducts. Goldblatt [10] observed that this was so for modal logic and the same quick little argument applies here. Recall that a class of structures is elementary iff both the class and its complement are closed under ultraproducts ([7], Corollary 6.1.16 (ii)^3^). Now, the complement of a class of Routley-Meyer frames definable by a relevant formula *ϕ* is always closed under ultraproducts. For it is the class of all models of a Σ11 sentence ∃*P*_1_,…,*P*_*n*_(*U* *p*_≤_(*P*_1_) ∧… ∧ *U* *p*_≤_(*P*_*n*_) ∧¬*T*_*x*_(*ϕ*)^*T*/*x*^) and Σ11 formulas are preserved under ultraproducts ([7], Corollary 4.1.14). Hence, we have proven the observation. Furthermore, following a similar argument to that in [21] for modal logic, we can actually show that ultrapowers suffice for a characterization of elementarity.

As an example of a relevant formula which defines an elementary class of frames we have $p_{0} \vee (p_{0} \rightarrow p_{1}) \vee {\dots } \vee \left (\bigwedge _{0 \leq i \leq n-1} p_{i} \rightarrow p_{n}\right )$. The intuitionistic counterpart of this formula on intuitionistic Kripke frames corresponds to a cardinality claim ([6], Proposition 2.40). The first order correspondent of the formula on Routley-Meyer frames is $\forall x_{1}, y_{1}, \dots , x_{n}, y_{n} \left (\bigwedge _{i=1}^{n} x_{i} \leq y_{i} \supset \bigvee _{1 \leq j \textless i \leq n } x_{i} \leq y_{j}\right )$. This can be seen as follows. Let $\mathfrak {F}$ be an arbitrary Routley-Meyer frame. Suppose first that $\mathfrak {F}$ satisfies our first order condition and that for some model *M* based on $\mathfrak {F}$, $M, T \nVdash p_{0} \vee (p_{0} \rightarrow p_{1}) \vee {\dots } \vee \left (\bigwedge _{0 \leq i \leq n-1} p_{i} \rightarrow p_{n}\right )$ for some *T* ∈ *O*. The latter means that for each number 1 ≤ *i* ≤ *n*, there are *x*_*i*_,*y*_*i*_ such that *x*_*i*_ ≤ *y*_*i*_, $M, x_{i} \Vdash \bigwedge _{0 \leq j \leq i-1} p_{j}$ but $M, y_{i} \nVdash p_{i}$. Now take *x*_*i*_,*y*_*j*_, *j*<*i* such that *x*_*i*_ ≤ *y*_*j*_. We have that $M, x_{i} \Vdash \bigwedge _{0 \leq j \leq i-1} p_{j}$ but $M, y_{j} \nVdash p_{j}$, which by the Hereditary Lemma implies that $M, x_{i} \nVdash p_{j} $, a contradiction. On the other hand, suppose $\forall x_{1}, y_{1}, \dots , x_{n}, y_{n} \left (\bigwedge _{i=1}^{n} x_{i} \leq y_{i} \supset \bigvee _{1 \leq j \textless i \leq n } x_{i} \leq y_{j}\right )$ fails in $\mathfrak {F}$, that is $\exists x_{1}, y_{1}, \dots , x_{n}, y_{n} \left (\left (\bigwedge _{i=1}^{n} x_{i} \leq y_{i} \right )\wedge \left (\bigwedge _{1 \leq j \textless i \leq n } x_{i} \nleq y_{j}\right )\right )$ holds in $\mathfrak {F}$. Without loss of generality, we may assume that for some *T* ∈ *O*, we have *x*_0_ = *T*,*T* = *y*_0_,*x*_1_,*y*_1_,…,*x*_*n*_,*y*_*n*_ is an enumeration such that *x*_*i*_ ≤ *y*_*j*_ implies that *i* ≤ *j*. Consider a valuation *V* on $\mathfrak {F}$ such that $V(p_{i}) = \{x : x \nleq y_{i}\}$ (this set is upward closed under ≤ by the transitivity of ≤). First thing to note is that $\langle \mathfrak {F}, V \rangle , T \nVdash p_{0}$ since ≤ is reflexive (which also implies that $\langle \mathfrak {F}, V \rangle , y_{i} \nVdash p_{i}$). But for any *i*>0, $\langle \mathfrak {F}, V \rangle , x_{i} \Vdash \bigwedge _{0 \leq j \leq i-1} p_{j}$, since otherwise for some *j*<*i*, *x*_*i*_ ≤ *y*_*j*_, which is impossible by assumption. Hence, $\mathfrak {F} \nVdash p_{0} \vee (p_{0} \rightarrow p_{1}) \vee {\dots } \vee \left (\bigwedge _{0 \leq i \leq n-1} p_{i} \rightarrow p_{n}\right )$, as desired.

The example in the above paragraph also serves as a point of comparison between the expressive power of relevant formulas on Routley-Meyer frames and intuitionistic formulas on Kripke frames. More illustrations of relevant formulas defining elementary classes will follow in Section 4, as applications of our Sahlqvist correspondence theorem.

One question that arises immediately is whether all relevant formulas have first order correspondents, i.e., define elementary classes. The next results show that this is not the case (though Section 4 will give a positive result). The formula witnessing this fact in the following theorem is roughly the analogue of the famous McKinsey axiom from modal logic (which was shown not to be elementary simultaneously by van Benthem [20] and Goldblatt [10]). We use a techinique due to van Benthem [20].

### **Theorem 5**

*The class of Routley-Meyer frames defined by the formula*

- ∼ (∼ *p* ∧ (**t**∨∼**t** → (*p* ∘ (**t**∨∼**t**)))) ∨ ((**t**∨∼**t**) ∘ (**t**∨∼**t** → *p*))

*is not elementary. In other words, the above formula has no first order correspondent.*

### *Proof*

Consider the structure $\mathfrak {F_{0}}= \langle W, R, *, \{q\} \rangle $, where

$$\begin{array}{@{}rcl@{}} W &=& \{q, s\} \cup \{q_{n} : n \in \omega \} \cup \{q_{n, i} : n \in \omega, i \in \{0, 1\}\} \cup \{r_{f} : f \in \{0, 1\}^{\omega}\},\\ R &=& \{\langle q, x, x \rangle : x \in W \} \cup \{\langle q_{n, i}, q_{n, j}, q_{n, j}\rangle : n \in \omega, i,j \in \{0, 1\}\} \\ &&\cup\{ \langle r_{f}, q_{n, f(n)}, q_{n, f(n)}\rangle : n \in \omega, f \in \{0, 1\}^{\omega}\} \cup \{\langle q_{n, i}, q_{n, i}, x\rangle : x \in W\\ &&\setminus \{q_{n} : n \in \omega \}, n \in \omega, i \in \{0, 1\}\} \cup \{\langle q_{n, i}, q_{n, i}, q_{n}\rangle : n \in \omega, i \in \{0, 1\} \}\\ && \cup \{\langle s, r_{f}, q \rangle : f \in \{0, 1\}^{\omega}\}x, \end{array} $$

and ∗ is the identity function. $\mathfrak {F_{0}}$ is trivially a Routley-Meyer frame, since in $\mathfrak {F_{0}}$, *a* ≤ *b* iff *a* = *b*. Note that any subset of W is upward closed with respect to ≤ due to this. It is easily seen that $\mathfrak {F_{0}}, w \Vdash \textbf {t} \vee \sim \textbf {t}$ for any *w* ∈ *W* by our definition of ∗, which basically makes ∼ collapse to boolean negation in $\mathfrak {F_{0}}$. Clearly, $\mathfrak {F_{0}}$ has uncountable cardinality. Also, we observe that over frames where ∗ is the identity (such as $\mathfrak {F_{0}}$), a formula like (M), of the form ∼ *ϕ* ∨ *ψ*, is essentially a material implication.

First, we show that (M) is valid in $\mathfrak {F_{0}}$. Let V be an arbitrary valuation on $\mathfrak {F_{0}}$. Suppose that $\langle \mathfrak {F_{0}}, V \rangle , q \Vdash \sim p \wedge (\textbf {t} \vee \sim \textbf {t} \rightarrow (p \circ (\textbf {t} \vee \sim \textbf {t})))$. This means that $\langle \mathfrak {F_{0}}, V \rangle , q \Vdash \sim p $ (which implies that $\langle \mathfrak {F_{0}}, V \rangle , q \nVdash p $ by our definition of ∗) and $\langle \mathfrak {F_{0}}, V \rangle , q \Vdash \textbf {t} \vee \sim \textbf {t} \rightarrow (p \circ (\textbf {t} \vee \sim \textbf {t}))$ (which implies that for all *w* ∈ *W*, $\langle \mathfrak {F_{0}}, V \rangle , w \Vdash p \circ (\textbf {t} \vee \sim \textbf {t})$, i.e., there are *v*_1_,*v*_2_ such that *R* *v*_1_ *v*_2_ *w* and $\langle \mathfrak {F_{0}}, V \rangle , v_{1} \Vdash p$). By the latter, for each *q*_*n*_ there must be some *q*_*n*,*i*_ such that $\langle \mathfrak {F_{0}}, V \rangle , q_{n, i} \Vdash p$ (for *q*,*q*_*n*_ cannot witness *p* ∘ (**t**∨∼**t**) since $\langle \mathfrak {F_{0}}, V \rangle , q \nVdash p$). Take *r*_*f*_ to be such that $\langle \mathfrak {F_{0}}, V \rangle , q_{n, f(n)} \Vdash p$ for any *n* ∈ *ω*. Then since *R* *s* *r*_*f*_ *q* and $\langle \mathfrak {F_{0}}, V \rangle , z_{f} \Vdash \textbf {t} \vee \sim \textbf {t} \rightarrow p$ (since in $\mathfrak {F_{0}}$, *R* *r*_*f*_ *x* *y* iff for some *n* ∈ *ω*, *x* = *q*_*n*,*f*(*n*)_ = *y*), we have that $\langle \mathfrak {F_{0}}, V \rangle , q \Vdash (\textbf {t} \vee \sim \textbf {t})\circ (\textbf {t} \vee \sim \textbf {t} \rightarrow p)$, as desired.

Now suppose (M) has a first order correspondent *ϕ*. By the above paragraph, $\mathfrak {F_{0}} \vDash \phi $. By the downward Löwenheim-Skolem theorem, there is a countable elementary substructure $\mathfrak {F_{0}}^{\prime }$ of $\mathfrak {F_{0}}$ including {*q*,*s*}∪{*q*_*n*_ : *n* ∈ *ω*}∪{*q*_*n*, *i*_ : *n* ∈ *ω*,*i* ∈{0, 1}} as a subset of its domain. We will show that (M) is not valid in $\mathfrak {F_{0}}^{\prime }$. This will produce a contradiction since $\mathfrak {F_{0}}^{\prime } \vDash \phi $, given that $\mathfrak {F_{0}}^{\prime }$ and $\mathfrak {F_{0}}$ are elementarily equivalent.

Being countable, $\mathfrak {F_{0}}^{\prime }$ must leave out an element *z*_*g*_ (*g* ∈{0, 1}^*ω*^) of W . Consider a valuation V on $\mathfrak {F_{0}}^{\prime }$ such that *V* (*p*) = {*q*_*n*,*g*(*n*)_ : *n* ∈ *ω*}. We note that $\langle \mathfrak {F_{0}}^{\prime }, V \rangle , q \nVdash p$, so $\langle \mathfrak {F_{0}}^{\prime }, V \rangle , q \Vdash \sim p$. Now, for any *q*_*n*_, since *R* *q*_*n*,*g*(*n*)_ *q*_*n*,*g*(*n*)_ *q*_*n*_, $\langle \mathfrak {F_{0}}^{\prime }, V \rangle , q_{n} \Vdash p \circ (\textbf {t} \vee \sim \textbf {t})$. Similarly if *w*≠*q*_*n*_ (*n* ∈ *ω*), $\langle \mathfrak {F_{0}}^{\prime }, V \rangle , w \Vdash p \circ (\textbf {t} \vee \sim \textbf {t})$. Hence, for all w in the domain of $\mathfrak {F_{0}}^{\prime }$, $\langle \mathfrak {F_{0}}^{\prime }, V \rangle , w \Vdash p \circ (\textbf {t} \vee \sim \textbf {t})$, which implies that $\langle \mathfrak {F_{0}}^{\prime }, V \rangle , q \Vdash \textbf {t} \vee \sim \textbf {t} \rightarrow (p \circ (\textbf {t} \vee \sim \textbf {t}))$. Finally, we want to show that $\langle \mathfrak {F_{0}}^{\prime }, V \rangle , q \nVdash (\textbf {t} \vee \sim \textbf {t})\circ (\textbf {t} \vee \sim \textbf {t} \rightarrow p)$. If *R* *x* *y* *q* either (1) *x* = *y* = *q* or (2) *x* = *y* = *q*_*n*,*i*_ for some *n* ∈ *ω*,*i* ∈{0,1}, or (3) *x* = *s* and *y* = *z*_*f*_ for some *z*_*f*_ in the domain of $\mathfrak {F_{0}}^{\prime }$. It suffices to show that in all three cases $\langle \mathfrak {F_{0}}^{\prime }, V \rangle , y \nVdash \textbf {t} \vee \sim \textbf {t} \rightarrow p$. If (1), since $\langle \mathfrak {F_{0}}^{\prime }, V \rangle , q \nVdash p$ and given that *R* *q* *q* *q*, $\langle \mathfrak {F_{0}}^{\prime }, V \rangle , y \nVdash \textbf {t} \vee \sim \textbf {t} \rightarrow p$. If (2), since *R* *q*_*n*,*i*_ *q*_*n*,*h*(*g*(*n*))_ *q*_*n*,*h*(*g*(*n*))_ (where *h* : {0,1}→{0,1} is the function such that *h*(0) = 1 and *h*(1) = 0) and $\langle \mathfrak {F_{0}}^{\prime }, V \rangle , q_{n, h(g(n))} \nVdash p$, we have that $\langle \mathfrak {F_{0}}^{\prime }, V \rangle , y \nVdash \textbf {t} \vee \sim \textbf {t} \rightarrow p$. If (3), *f*≠*g*, so they differ in their value for some n, hence, *q*_*n*,*f*(*n*)_≠*q*_*n*,*g*(*n*)_, which implies that $\langle \mathfrak {F_{0}}^{\prime }, V \rangle , q_{n, f(n)} \nVdash p$. Given that *R* *r*_*f*_ *q*_*n*,*f*(*n*)_ *q*_*n*,*f*(*n*)_, we see that $\langle \mathfrak {F_{0}}^{\prime }, V \rangle , y \nVdash \textbf {t} \vee \sim \textbf {t} \rightarrow p$, as desired. □

Next we provide a much easily graspable non-elementary class of Routley-Meyer frames which is definable in the language of relevant logic. For the conjunction of conditions (i) and (ii) in Proposition 6 is not expressible in first order logic as will be seen through a compactness argument. This time we build a relevant logic analogue of the well-known Löb axiom.

Put *R* ^*#*^ *x* *y*= _*df*_∃*z*(*R* *x* *y* *z* ∨ *R* *x* *z* *y*) and ▪*p*= _*df*_(*p*∨∼ *p* → *p*) ∧ (∼ *p* → *p*∧∼ *p*). Observe that for any frame $\mathfrak {F}$, and world *x* ∈ *W*, *R* ^*#*^ *T* *x* holds, so $\mathfrak {F} \vDash \forall x (R^{\#}Tx)$. In any frame $\mathfrak {F}$ where ∀*x*(*x* ^∗^≤ *x* ∧ *x* ≤ *x* ^∗^), using the Hereditary Lemma, we see that a formula of the form ∼ *ϕ* ∨ *ψ* behaves essentially as a material implication in a classical language at the level of models based on $\mathfrak {F}$. Also, for any valuation *V* in any such frame $\mathfrak {F}$, $\langle \mathfrak {F}, V \rangle , w \Vdash \blacksquare p $ iff for all *x*,*y* such that *R* *w* *x* *y*, $\langle \mathfrak {F}, V \rangle , x\Vdash p $ and $\langle \mathfrak {F}, V \rangle , y\Vdash p $ iff for all *x* such that *R* ^*#*^ *w* *x*, $\langle \mathfrak {F}, V \rangle , x\Vdash p $.

Now, for any frame $\mathfrak {F}$, $\mathfrak {F} \Vdash (p \wedge \sim p \rightarrow q) \wedge (q \rightarrow p \vee \sim p)$ iff $\mathfrak {F} \vDash \forall x (x^{*} \leq x \wedge x \leq x^{*})$.

### **Proposition 6**

$\mathfrak {F} \Vdash (p \wedge \sim p \rightarrow q) \wedge (q \rightarrow p \vee \sim p) \wedge ((\blacksquare (\blacksquare p \supset p) \wedge p) \supset \blacksquare p)$ *iff (i)* $\mathfrak {F} \vDash \forall x (x^{*} \leq x \wedge x \leq x^{*})$ *and there is* *T* ∈ *O* *such that (ii) there is no infinite sequence of worlds* *T* = *s*_0_,*s*_1_,*s*_2_…*such that* $s_{0} \nleq s_{i} (0 \textless i \textless \omega )$ *and* *R* ^*#*^ *s*_0_ *s*_1_,*R* ^*#*^ *s*_1_ *s*_2_,*R* ^*#*^ *s*_2_ *s*_3_,…*and (iii) for any* *x*,*y* *,* *R* ^*#*^ *T* *x* *and* *R* ^*#*^ *x* *y* *implies that* *R* ^*#*^ *T* *y* *.*

### *Proof*

Let $\mathfrak {F}$ be an arbitrary Routley-Meyer frame. We have that if (i) holds, the validity of ▪(▪*p* ⊃ *p*) ∧ *p* ⊃ ▪*p* implies (ii). For suppose (ii) fails, then there is an infinite sequence of worlds $T=s_{0} \nleq s_{1}, s_{2} \dots $ such that *R* ^*#*^ *s*_0_ *s*_1_, *R* ^*#*^ *s*_1_ *s*_2_, *R* ^*#*^ *s*_2_ *s*_3_,… Now take any valuation V based on $\mathfrak {F}$ such that $V(p)=\{w : w \nleq s_{i}, 0 \textless i\textless \omega \}$. By transitivity of ≤, *V* (*p*) is upwards closed under ≤. For each *s*_*i*_, *s*_*i*_ ≤ *s*_*i*_, so $\langle \mathfrak {F}, V \rangle , s_{i} \nVdash p$. Hence, $\langle \mathfrak {F}, V \rangle , T \nVdash \blacksquare p$. Also, by assumption, $T \nleq s_{i}$ (0<*i*<*ω*), which mean that $\langle \mathfrak {F}, V \rangle , T \Vdash p$. Now let *R* ^*#*^ *T* *v* and suppose that $\langle \mathfrak {F}, V \rangle , v \Vdash \blacksquare p$ but $\langle \mathfrak {F}, V \rangle , v \nVdash p$. The latter means that *v* ≤ *s*_*i*_ for some 0<*i*<*ω*, however since $\langle \mathfrak {F}, V \rangle , s_{i+1} \nVdash p$ and *R* ^*#*^ *s*_*i*_ *s*_*i*+1_, it must be that $\langle \mathfrak {F}, V \rangle , s_{i} \nVdash \blacksquare p$, and by the Hereditary Lemma, $\langle \mathfrak {F}, V \rangle , v \nVdash \blacksquare p$. Hence, $\langle \mathfrak {F}, V \rangle , T \Vdash \blacksquare (\blacksquare p \supset p)$. Similarly, if (iii) fails, we have some *x*,*y* such that *R* ^*#*^ *T* *x* and *R* ^*#*^ *x* *y* but not *R* ^*#*^ *T* *y*. Just consider a valuation V such that $V(p)=\{w : w \nleq x, y \}$. *V* (*p*) turns out to be upwards closed under ≤ due to the transitivity of ≤. This concludes the left to right direction of the proposition.

For the converse suppose $\langle \mathfrak {F}, V \rangle , T \nVdash \blacksquare (\blacksquare p \supset p) \wedge p \supset \blacksquare p$. If (i) holds, one can build the desired sequence to falsify (ii) by taking x such that *R* ^*#*^ *T* *x* while $\langle \mathfrak {F}, V \rangle , x \nVdash p$ and applying $\langle \mathfrak {F}, V \rangle , T \Vdash \blacksquare (\blacksquare p \supset p)$ in conjunction with (iii). □

To see that the conjunction of conditions (i) and (ii) in Proposition 6 is not a first order property, let us suppose it is to derive a contradiction. Say *ϕ* is the first order formula expressing (i) and (ii). Expand the correspondence language by adding a constant *c*_*i*_ for each *i*>0. Let Δ be the collection of first order formulas axiomatizing our class of Routley-Meyer frames and Θ the set of first order formulas {*O*(*T*),*R* ^*#*^ *T* *c*_1_,*R* ^*#*^ *c*_*i*_ *c*_*i*+1_ : 0<*i*<*ω*}. For each *n*>0, take the frame $\mathfrak {F}_{n}$ where *W* = {*k* : *k* ≤ *n*}, *R* = {〈0,*i*, *i*〉 : *i* ≤ *n*}∪{〈*j*,*j* + 1,*j* + 1〉 : *j*<*n*}, *T* is simply the number 0 while *O* = {0} and ∗ the identity. Each finite subset of {*ϕ*}∪ Δ ∪ Θ has a model in $\mathfrak {F}_{n}$ for sufficiently large *n*. By compactness, the whole thing must have a model, which is impossible since *ϕ* forbids Θ from being true by assumption.

So we have seen that at the level of frames, the language of relevant logic can go beyond the expressive power of first order logic. More precisely, the fragment of monadic second order logic corresponding to the language of relevant logic over frames can express some non-first order concepts. Does it contain first order logic, though? We will give next an easy argument that it does not (though there was never a reason to believe that it did). This shows that the “relevant fragment” of monadic second order logic is indeed incomparable with first order logic in terms of expressive power.

### **Definition 2**

Let *M* = 〈*W*, *R*, ∗, *O*, *V* 〉 and *N* = 〈*W* ^′^,*R* ^′^,∗^′^, *O* ^′^, *V* ^′^〉 be models for a basic relevant language with absurdity L. A map *f* : *W*→*W* ^′^ is called a bounded morphism if it satisfies the following:

- (i) x and *f*(*x*) satisfy the same propositional variables.
- (ii) *R* *x* *y* *z* only if *R* ^′^ *f*(*x*)*f*(*y*)*f*(*z*).
- (iii) $f(x)^{*^{\prime }} = f(x^{*}) $.
- (iv) $R^{\prime }f(x)y^{\prime }z^{\prime }$ only if there are *y*,*z* such that *R* *x* *y* *z* and *f*(*y*) = *y* ^′^ and *f*(*z*) = *z* ^′^.
- (v) *f*(*x*) ∈ *O* ^′^ for *x* ∈ *O*
- (vi) *R* ^′^ *y* ^′^ *z* ^′^ *f*(*x*) only if there are *y*,*z* such that *R* *y* *z* *x* and *f*(*y*) = *y* ^′^ and *f*(*z*) = *z* ^′^.
- (vii) *R* ^′^ *y* ^′^ *z* ^′^ *f*(*x*) for *y* ^′^,*z* ^′^∈ *O* ^′^ only if there are *y*,*z* ∈ *O* such that *R* *y* *z* *x* and *f*(*y*) = *y* ^′^ and *f*(*z*) = *z* ^′^.

### **Lemma 7**

*Let* *M*,*N* *be two models and* f *a bounded morphism from* M *into* N*. Then, for any world* w *of* M *and relevant formula* *ϕ* *,* $M, w \Vdash \phi $ *iff* $N, f(w) \Vdash \phi $ *.*

### *Proof*

Routine induction on formula complexity. □

When one omits condition (i) in Definition 2, we might speak of a bounded morphism from a frame $\mathfrak {F}$ into a frame $\mathfrak {F}^{\prime }$.

### **Proposition 8**

*If there is a bounded morphism* f *from a frame* $\mathfrak {F} = \langle W, R, *, O \rangle $ *into a frame* $\mathfrak {F}^{\prime } = \langle W^{\prime }, R^{\prime }, *^{\prime }, O^{\prime }\rangle $ *,* *then* $\mathfrak {F} \Vdash \phi $ *implies* $\mathfrak {F}^{\prime } \Vdash \phi $ *for any relevant formula* *ϕ* *.*

### *Proof*

Suppose $\mathfrak {F}^{\prime } \nVdash \phi $. So there is a valuation *V* ^′^ such hat $\langle \mathfrak {F}, V^{\prime } \rangle , T \nVdash \phi $ for some *T* ∈ *O*. Consider now the valuation V such that *V* (*p*) = {*x* ∈ *W* : *f*(*x*) ∈ *V* ^′^(*p*)} for any p. Note that *V* (*p*) is closed under ≤ in $\mathfrak {F}$. For suppose *x* ∈ *V* (*p*) (i.e., *f*(*x*) ∈ *V* ^′^(*p*)) and *x* ≤ *y* in $\mathfrak {F}$, which by conditions (ii) and (v) in Definition 2 and our assumption, implies that *f*(*x*) ≤ *f*(*y*). Hence, *f*(*y*) ∈ *V* ^′^(*p*), which means that *y* ∈ *V* (*p*). So f is now a bounded morphism from the model $\langle \mathfrak {F}, V \rangle $ into the model $\langle \mathfrak {F}^{\prime }, V^{\prime } \rangle $, which, using Proposition 8, means that $\mathfrak {F} \nVdash \phi $. □

Finally, let $\mathfrak {F}_{1}$ be the frame 〈*W*,*R*,∗,{*s*,*t*}〉 where *W* = {*s*,*t*}, *R* = *W* × *W* × *W* and ∗ = {〈*t*,*t*〉,〈*s*,*t*〉}. On the other hand, let $\mathfrak {F}_{2}$ be the frame 〈*W* ^′^,*R* ^′^,∗^′^,{*s*}〉 where *W* ^′^ = {*s*}, *R* = *W* ^′^× *W* ^′^× *W* ^′^ and ∗ is the identity. There is only one function *f* : *W*→*W* ^′^. The function *f* is a bounded morphism from $\mathfrak {F}_{1}$ onto $\mathfrak {F}_{2}$ as it is easy to check. The first order property ∃*x*(*O* *x* ∧ *x*≠*x* ^∗^) (where the denotation of *T* is *s* in both $\mathfrak {F}_{1}$ and $\mathfrak {F}_{2}$) holds at $\mathfrak {F}_{1}$ but fails at $\mathfrak {F}_{2}$, so by Proposition 8, ∃*x*(*O* *x* ∧ *x*≠*x* ^∗^) is not definable by a relevant formula.

## A Sahlqvist Correspondence Theorem

In this section, we prove the result promised in Section 1 using the groundwork layed out in Section 2. In the proof we use lambda terms as in [3], which are to be understood as predicate constants (i.e., the denotation of the lambda term *λ* *u*.(*ϕ*) is fixed by the set of worlds satisfying *ϕ*).

### **Lemma 9**

*Let* *ϕ*, *𝜃*, *ψ* *and* *χ* *be relevant formulas such that* *𝜃* *contains no propositional variable and* *ϕ* *and* *χ* *have no propositional variable in common. Suppose* *ϕ*,*ψ* *and* *χ* *have first order frame correspondents. Then* *𝜃* → *ϕ*,*ϕ* ∧ *ψ* *and* *ϕ* ∨ *χ* *have first order correspondents.*

### *Proof*

Suppose *ϕ* ^′^,*ψ* ^′^,*χ* ^′^ are the first order local correspondents of *ϕ*,*ψ* and *χ* respectively.

For (*𝜃* → *ϕ*), supposing that all its propositional variables appear in the list *p*_1_,…, *p*_*n*_, we have that

$$\begin{array}{llll} \!\mathfrak{F}, w \!\Vdash\! \theta \!\rightarrow\! \phi & \text{iff} & \mathfrak{F} \vDash \forall P_{1}, \dots, P_{n} \forall x, y (\bigwedge_{i<n+1} Up_{\leq}(P_{i}) \wedge Rxyz \wedge T_{x}(\theta) \supset T_{x}(\phi)^{z/x}) [w]\\ & \text{iff} & \mathfrak{F} \vDash \forall P_{1}, \dots, P_{n} \forall x, y (Rxyz \wedge T_{x}(\theta) \supset (\bigwedge_{i<n+1} Up_{\leq}(P_{i}) \supset T_{x}(\phi)^{z/x})) [w]\\ & \text{iff} & \mathfrak{F} \vDash \forall x, y (Rxyz \wedge T_{x}(\theta) \supset \forall P_{1}, \dots, P_{n} (\bigwedge_{i<n+1} Up_{\leq}(P_{i}) \supset T_{x}(\phi)^{z/x})) [w]\\ & \text{iff} & \mathfrak{F} \vDash \forall x, y (Rxyz \wedge T_{x}(\theta) \supset \phi^{\prime}) [w].\\ \end{array} $$

For (*ϕ* ∧ *ψ*), supposing that all its propositional variables appear in the list *p*_1_,…,*p*_*n*_, we have that

$$\begin{array}{llll} \mathfrak{F}, w \Vdash \phi \wedge \psi & \text{iff} & \mathfrak{F} \vDash \forall P_{1}, \dots, P_{n} (\bigwedge_{i<n+1} Up_{\leq}(P_{i}) \supset T_{x}(\phi) \wedge T_{x}(\psi)) [w]\\ & \text{iff} & \mathfrak{F} \vDash \forall P_{1}, \dots, P_{n} ((\bigwedge_{i<n+1} Up_{\leq}(P_{i}) \supset T_{x}(\phi)) \wedge \\ & & (\bigwedge_{i<n+1} Up_{\leq}(P_{i}) \supset T_{x}(\psi))) [w]\\ & \text{iff} &\mathfrak{F} \vDash \forall P_{1}, \dots, P_{n}(\bigwedge_{i<n+1} Up_{\leq}(P_{i}) \supset T_{x}(\phi)) \wedge\\ & & \forall P_{1}, \dots, P_{n}(\bigwedge_{i<n+1} Up_{\leq}(P_{i}) \supset T_{x}(\psi)) [w]\\ & \text{iff} & \mathfrak{F} \vDash \phi^{\prime} \wedge \psi^{\prime}[w].\\ \end{array} $$

For (*ϕ* ∨ *χ*), $\mathfrak {F}, w \Vdash \phi \vee \chi $ iff $\mathfrak {F}, w \Vdash \phi $ or $\mathfrak {F}, w \Vdash \chi $. Right to left is obvious. For the converse suppose that $\mathfrak {F}, w \Vdash \phi \vee \chi $, $\mathfrak {F}, w \nVdash \phi $ and $\mathfrak {F}, w \nVdash \chi $. Then there are *V*_1_,*V*_2_ such that $\langle \mathfrak {F}, V_{1} \rangle , w \nVdash \phi $ and $\langle \mathfrak {F}, V_{2} \rangle , w \nVdash \chi $. It is easy to show by induction on formula complexity that for any two valuations *V*,*V* ^′^ on a frame $\mathfrak {F}$ such that *V* (*p*) = *V* ^′^(*p*) for all propositional variables *p* appearing in a formula *ϕ*, $\langle \mathfrak {F}, V \rangle , w \nVdash \phi $ iff $\langle \mathfrak {F}, V^{\prime } \rangle , w \nVdash \phi $ for any world *w* of $\mathfrak {F}$. Say *p*_1_,…,*p*_*n*_ and *q*_1_,…,*q*_*m*_ are the propositional variables appearing in *ϕ*,*χ* respectively. Take a valuation *V*_3_ such that *V*_3_(*p*_*i*_) = *V*_1_(*p*_*i*_) while *V*_3_(*q*_*j*_) = *V*_2_(*q*_*j*_). It follows that $\langle \mathfrak {F}, V_{3} \rangle , w \nVdash \phi $ and $\langle \mathfrak {F}, V_{3} \rangle , w \nVdash \chi $, contradicting our assumption that $\mathfrak {F}, w \Vdash \phi \vee \chi $. Finally, $\mathfrak {F}, w \Vdash \phi $ or $\mathfrak {F}, w \Vdash \chi $ iff $\mathfrak {F} \vDash \phi ^{\prime } [w]$ or $\mathfrak {F} \vDash \chi ^{\prime } [w]$ iff $\mathfrak {F} \vDash \phi ^{\prime } \vee \chi ^{\prime } [w]$. □

### **Definition 3**

A formula *ϕ* → *ψ* is called a relevant Sahlqvist implication if *ψ* is positive while *ϕ* is a formula built up from propositional atoms, double negated atoms (i.e., formulas of the form ∼∼ *p*), negative formulas, the constant **t** and implications of the form **t** → *p* (for any propositional variable p) using only the connectives ∨,∧ and ∘.

### *Example 10*

*p*∧∼ *p* → *q*,∼∼ *p* → *p*,*p* ∘ *q* → *p* ∧ *q*,((*p* ∘ *q*) ∨ (**t** → *p*)) → *p* ∨ *q*, *p* → (**t**∨∼**t** → *p*) and (**t** → *p*) → *p* are all relevant Sahlqvist implications according to Definition 3, while (*p* → (*p* → *q*)) → (*p* → *q*) is not since, for one thing, *T*_*x*_(*p* → *q*) is not a positive formula.

### **Lemma 11**

*Every relevant Sahlqvist implication has a local first order correspondent on* *Routley-Meyer frames.*

### *Proof*

Let *ϕ* → *ψ* be a relevant Sahlqvist implication. We consider the local frame correspondent

- (T) ∀*P*_1_,…,*P*_*n*_∀*y*,*z*(*U* *p*_≤_(*P*_1_) ∧… ∧ *U* *p*_≤_(*P*_*n*_) ∧ *R* *x* *y* *z* ∧ *T*_*x*_(*ϕ*)^*y*/*x*^ ⊃ *T*_*x*_(*ψ*)^*z*/*x*^).

Renaming variables we can make sure that no two quantifiers bind the same variable and that x remains free. One can abbreviate *T*_*x*_(*ψ*)^*z*/*x*^, which is a positive formula, as POS.

Using the classical principles of associativity, distributivity and the well-known equivalences

- (∃*x* *𝜃*(*x*) ∧ *σ*) ≡∃*x*(*𝜃*(*x*) ∧ *σ*),
- (∃*x* *𝜃*(*x*) ⊃ *σ*) ≡∀*x*(*𝜃*(*x*) ⊃ *σ*),
- (*𝜃* ∨ *δ* ⊃ *σ*) ≡ ((*𝜃* ⊃ *σ*) ∧ (*δ* ⊃ *σ*)),
- ∀*P*_1_,…,*P*_*n*_∀*x*_1_,…, *x*_*k*_(*𝜃*∧*σ*) ≡ ((∀*P*_1_,…, *P*_*n*_∀*x*_1_,…, *x*_*k*_…*𝜃*)∧(∀*P*_1_,…, *P*_*n*_∀*x*_1_,…, *x*_*k*_…*σ*)),

by “pulling out” the fusions in the antecedent of (T), we will obtain a conjunction of formulas of the form:

- (1) ∀*P*_1_,…,*P*_*n*_∀*x*_1_,…,*x*_*k*_(*U* *p*_≤_(*P*_1_)∧…∧*U* *p*_≤_(*P*_*n*_)∧REL∧(DN)AT∧IMP∧NEG ⊃POS).

Here, REL is a conjunction of atomic first order expressions involving only the ternary predicate R, (DN)AT is a conjunction of translations of (double negated) propositional variables, IMP is a conjunction of translations of formulas of the form **t** → *p*_*i*_, that is, formulas of the form ∀*y*,*z*(*R* *x* *y* *z* ∧∃*b*(*O* *b* ∧ *b* ≤ *y*) ⊃ *P*_*i*_ *z*), while NEG is a conjunction of negative formulas. Our purpose will be to eliminate all the second order quantifiers in (1).

Next we observe that one may assume that any unary predicate (corresponding to a propositional variable) appearing in POS, appears also in the antecedent of (1). For suppose not, that is, there a unary predicate *P*_*i*_ which appears only in POS. Take

- (2) $[\lambda u.(u \nleq u)/P_{i}]\forall P_{1}, {\dots } , P_{i-1}, P_{i+1}, {\dots } , P_{n} \forall x_{1}, {\dots } , x_{k} (Up_{\leq }(P_{1}) \\ \quad \wedge {\dots } \wedge Up_{\leq }(P_{n}) \wedge \text {REL} \wedge \text {(DN)AT} \wedge \text {IMP} \wedge \text {NEG} \supset \text {POS}),$

the formula resulting by instantiating the quantifier ∀*P*_*i*_ to the particular instance $\lambda u.(u \nleq u)$. Note that the extension of $\lambda u.(u \nleq u)$ is empty on any Routley-Meyer frame (by p.1) and that, moreover, $Up_{\leq }(\lambda u.(u \nleq u))$ holds trivially. Now assume that (2) to show that (1) holds. Take arbitrary *P*_1_…*P*_*n*_ and suppose that the antecedent of (1) holds. By (2), it follows that $[\lambda u.(u \nleq u)/P_{i}]$POS, but by Lemma 3, [*P*_*i*_]POS must be the case. Hence, (1). So (1) and (2) are actually equivalent.

It is easy to see that (1) is equivalent to

- (3) ∀*P*_1_,…,*P*_*n*_∀*x*_1_,…,*x*_*k*_(*U* *p*_≤_(*P*_1_)∧…∧*U* *p*_≤_(*P*_*n*_)∧REL∧(DN)AT∧IMP ⊃¬NEG∨POS).

Observe that ¬NEG ∨POS is a positive formula.

Suppose *π*_1_(*x*_*i*1_),…,*π*_*k*_(*x*_*ik*_) are all the conjuncts of (DN)AT and IMP in the antecedent of (3) in which the unary predicate *P*_*i*_ occurs. Then if *π*_*j*_(*x*_*ij*_) appears in one of the conjuncts in IMP, it must be a formula of the form ∀*y* *z*(*R* *x*_*ij*_ *y* *z* ∧∃*b*(*O* *b* ∧ *b* ≤ *y*) ⊃ *P*_*i*_ *z*), in which case we define *σ*(*π*_*j*_(*x*_*ij*_)) = *λ* *u*.(∃*y* *z*(*R* *x*_*ij*_ *y* *z* ∧∃*b*(*O* *b* ∧ *b* ≤ *y*) ∧ *z* ≤ *u*)). On the other hand if *π*_*j*_(*x*_*ij*_) appears in one of the conjuncts in (DN)AT we put *σ*(*π*_*j*_(*x*_*ij*_)) = *λ* *u*.(*x*_*ij*_ ≤ *u*) in case *π*_*j*_(*x*_*ij*_) = *P*_*i*_ *x*_*ij*_ and *σ*(*π*_*j*_(*x*_*ij*_)) = *λ* *u*.(*x* *i* *j*∗∗≤ *u*) in case *π*_*j*_(*x*_*ij*_) = ¬¬*P*_*i*_ *x* *i* *j*∗∗. Note that *σ* is a well-defined function and that for any *π*_*j*_(*x*_*ij*_), if *π*_*j*_(*x*_*ij*_)[*w*] then ∀*y*(*σ*(*π*_*j*_(*x*_*ij*_)(*y*) ⊃ *P*_*i*_ *y*)[*w*]. Next define *δ*(*P*_*i*_) = *λ* *u*.(*σ*(*π*_1_(*x*_*i*1_))(*u*) ∨… ∨ *σ*(*π*_*k*_(*x*_*ik*_))(*u*)). Now, if (DN)AT[*w* *w*_1_…*w*_*k*_] and IMP[*w* *w*_1_…*w*_*k*_] then ∀*u*(*δ*(*P*_*i*_)(*u*) ⊃ *P*_*i*_ *u*)[*w* *w*_1_…*w*_*k*_].

At this point we should note that *U* *p*_≤_(*δ*(*P*_*i*_)) holds for any *P*_*i*_. This is so because each *σ*(*π*_*j*_(*x*_*ij*_))(*u*) is upward closed under ≤, and any union of upward closed sets under ≤ is also upward closed under ≤. Moreover, by the reflexivity of ≤, [*δ*(*P*_1_)/*P*_1_…*δ*(*P*_*n*_)/*P*_*n*_](DN)AT and [*δ*(*P*_1_)/*P*_1_…*δ*(*P*_*n*_)/*P*_*n*_]IMP will hold trivially. So, from

- (4) [*δ*(*P*_1_)/*P*_1_…*δ*(*P*_*n*_)/*P*_*n*_]∀*x*_1_,…,*x*_*k*_(*U* *p*_≤_(*P*_1_)∧…∧*U* *p*_≤_(*P*_*n*_)∧REL∧(DN)AT∧IMP ⊃¬NEG∨POS),

we end up with the equivalent formula

- (5) ∀*x*_1_,…,*x*_*k*_(REL ⊃ [*δ*(*P*_1_)/*P*_1_…*δ*(*P*_*n*_)/*P*_*n*_](¬NEG ∨POS)).

Given that any unary predicate appearing in POS also appears in the antecedent of (4), (5) is a first order formula in the signature of Routley-Meyer frames, which contains *R*,∗, and O as the only non-logical symbols.

We have seen that (1) implies (5). All that is left is to show that (5) implies (1). Recall that (1) is equivalent to (3). Thus it suffices to prove that (5) implies (3). Assume (5), let *P*_1_,…,*P*_*n*_ be arbitrary and suppose further that

$$Up_{\leq}(P_{1}) \wedge {\dots} \wedge Up_{\leq}(P_{n}) \wedge \text{REL} \wedge \text{(DN)AT} \wedge \text{IMP}[ww_{1} {\dots} w_{k}], $$

so, by (5), we obtain that

$$[\delta(P_{1})/P_{1} {\dots} \delta(P_{n})/P_{n}] (\neg \text{NEG} \vee \text{POS})[ww_{1} {\dots} w_{k}]. $$

But recall that since (DN)AT[*w* *w*_1_…*w*_*k*_] and IMP[*w* *w*_1_…*w*_*k*_], it must be the case that ∀*u*(*δ*(*P*_*i*_)(*u*) ⊃ *P*_*i*_ *u*)[*w* *w*_1_…*w*_*k*_]. Hence, by Lemma 3,

$$(\neg \text{NEG} \vee \text{POS})[ww_{1} {\dots} w_{k}], $$

which proves (3). □

The procedure in the above proof is better understood by working out some examples, which we will do next for the benefit of the reader.

### *Example 12*

By the Sahlqvist-van Benthem algorithm, the following list of equivalences must hold:

$$\begin{array}{llll} \mathfrak{F}, w \Vdash p \wedge \sim p \rightarrow q & \text{iff} & \mathfrak{F} \vDash \forall P, Q \forall y, z (Up_{\leq}(P) \wedge Up_{\leq}(Q)\\ & & \wedge Rxyz \wedge Py \wedge \neg Py^{*} \supset Qz)[w]\\ & \text{iff} & x\mathfrak{F} \vDash \forall P \forall y, z (Up_{\leq}(P) \wedge Up_{\leq}(\lambda u. (u \nleq u)) \wedge Rxyz\\ & & \wedge Py \wedge \neg Py^{*} \supset \lambda u. (u \nleq u)(z))[w]\\ & \text{iff} & \mathfrak{F} \vDash \forall P \forall y, z (Up_{\leq}(P) \wedge Rxyz \wedge Py \wedge \neg Py^{*} \supset z \nleq z)[w]\\ & iff & \mathfrak{F} \vDash \forall P \forall y, z (Up_{\leq}(P) \wedge Rxyz \wedge Py \supset Py^{*} \vee z \nleq z)[w]\\ & \text{iff} & \mathfrak{F} \vDash \forall y, z (Up_{\leq}(\lambda u. (y \leq u)) \wedge Rxyz\\ & & \wedge \lambda u. (y \leq u)(y) \supset \lambda u. (y \leq u)(y^{*}) \vee z \nleq z)[w]\\ & \text{iff} & \mathfrak{F} \vDash \forall y, z (Rxyz \wedge y \leq y \supset y \leq y^{*} \vee z \nleq z)[w]\\ & \text{iff} & \mathfrak{F} \vDash \forall y, z (Rxyz \supset y \leq y^{*} \vee z \nleq z)[w]. \\ \end{array} $$

It is easy to check that this correspondence is accurate. First, if $\mathfrak {F} \vDash \forall y, z (Rxyz \supset y \leq y^{*} \vee z \nleq z)[w]$ and *M* is any model based on $\mathfrak {F}$, if *R* *w* *y* *z* and $M, y \Vdash p \wedge \sim p$ either $z \nleq z$ or $M, y \Vdash p$ and $M, y \nVdash p$ (contraposing the Hereditary Lemma). So, $M, w \Vdash p \wedge \sim p \rightarrow q$ as desired. Conversely, if $\mathfrak {F} \nvDash \forall y, z (Rxyz \supset y \leq y^{*} \vee z \nleq z)[w]$, $\mathfrak {F} \nvDash \exists y, z (Rxyz \wedge y \nleq y^{*} \wedge z \leq z)[w]$, i.e., $\mathfrak {F} \nvDash \exists y, z (Rxyz \wedge y \nleq y^{*} )[w]$. Take a valuation *V* on $\mathfrak {F}$ such that *V* (*p*) = {*x* : *y* ≤ *x*} and $V(q) = \{x : x \nleq z\}$ (note that these two sets are upward closed under ≤ by transitivity of ≤). *V* suffices to show that $\mathfrak {F}, w \nVdash p \wedge \sim p \rightarrow q$. Note that from this correspondence, we obtain that $\mathfrak {F} \Vdash p \wedge \sim p \rightarrow q$ iff $\mathfrak {F} \vDash \forall y, z (y \leq z \supset y \leq y^{*} )$ iff $\mathfrak {F} \vDash \forall y (y \leq y^{*})$.

### *Example 13*

By the Sahlqvist-van Benthem algorithm,

$$\begin{array}{llll} \mathfrak{F}, w \Vdash \sim \sim p \rightarrow p &\text{iff} & \mathfrak{F} \vDash \forall P \forall y, z (Up_{\leq}(P) \wedge Rxyz \wedge \neg \neg Py^{**} \supset Pz)[w]\\ & \text{iff} & \mathfrak{F} \vDash \forall y, z (Up_{\leq}(\lambda u. (y^{**} \leq u)) \wedge Rxyz \\ & & \wedge \neg \neg \lambda u. (y^{**} \leq u)(y^{**}) \supset \lambda u. (y^{**} \leq u)(z))[w]\\ & \text{iff} & \mathfrak{F} \vDash \forall y, z (Rxyz \wedge y^{**} \leq y^{**} \supset y^{**} \leq z)[w]\\ &\text{iff} & \mathfrak{F} \vDash \forall y, z (Rxyz \supset y^{**} \leq z)[w].\\ \end{array} $$

Next we establish that the correspondence is correct. Suppose that $\mathfrak {F} \vDash \forall y, z (Rxyz \supset y^{**} \leq z)[w]$, so, using the Hereditary Lemma, $\mathfrak {F}, w \Vdash \sim \sim p \rightarrow p$. On the other hand if $\mathfrak {F} \nvDash \forall y, z (Rxyz \supset y^{**} \leq z)[w]$, i.e., $\mathfrak {F} \vDash \exists y, z (Rxyz \wedge y^{**} \nleq z)[w]$. Then any valuation *V* such that $V(p) = \{x : x \nleq z\}$ guarantees that $\langle \mathfrak {F}, V \rangle , z \nvDash p$ and $\langle \mathfrak {F}, V \rangle , y^{**} \vDash p$, so $\mathfrak {F}, w \nVdash \sim \sim p \rightarrow p$.

The above condition reduces to ∀*x*(*x* ^∗∗^≤ *x* ∧ *x* ≤ *x* ^∗∗^) when we consider validity with respect to all the worlds in *O* of the frame. This is not the same in general as ∀*x*(*x* = *x* ^∗∗^) which is the condition usually required to validate ∼∼ *p* → *p*. However, it is certainly the case that, using the construction from Theorem 5 [12], by restricting our attention to just the frames where ≤ is antisymmetric, we get exactly the same set of validities as in the general case.

### *Example 14*

By the Sahlqvist-van Benthem algorithm,

$$\begin{array}{llll} \mathfrak{F}, w \!\Vdash\! p \circ q \!\rightarrow\! p \wedge q &\text{iff} & \mathfrak{F} \vDash \forall P, Q \forall y, z (Up_{\leq}(P) \wedge Up_{\leq}(Q) \wedge Rxyz \\ && \wedge \exists u_{1}u_{2} (Ru_{1}u_{2}y \wedge Pu_{1} \wedge Qu_{2}) \supset Pz \wedge Qz)[w]\\ & \text{iff}& \mathfrak{F} \vDash \forall P, Q \forall y, z, u_{1}, u_{2} (Up_{\leq}(P) \wedge Up_{\leq}(Q) \wedge Rxyz \\ && \wedge Ru_{1}u_{2}y \wedge Pu_{1} \wedge Qu_{2} \supset Pz \wedge Qz)[w]\\ & \text{iff}& \mathfrak{F} \vDash \forall y, z, u_{1}, u_{2} (Up_{\leq}(\lambda u_{3}.(u_{1} \leq u_{3})) \wedge Up_{\leq}(\lambda u_{3}.(u_{2} \leq u_{3}))\\ && \wedge Rxyz \wedge Ru_{1}u_{2}y \wedge \lambda u_{3}.(u_{1} \leq u_{3}) (u_{1})\\ && \wedge \lambda u_{3}.(u_{2} \leq u_{3}) (u_{2}) \supset \lambda u_{3}.(u_{1} \leq u_{3}) (z) \wedge \lambda u_{3}.(u_{2} \leq u_{3})(z))[w]\\ & \text{iff} & \mathfrak{F} \vDash \forall y, z, u_{1}, u_{2} (Rxyz \wedge Ru_{1}u_{2}y \wedge u_{1} \leq u_{1}\\ && \wedge u_{2} \leq u_{2} \supset u_{1} \leq z \wedge u_{2} \leq z)[w]\\ &\text{iff}& \mathfrak{F} \vDash \forall y, z, u_{1}, u_{2} (Rxyz \wedge Ru_{1}u_{2}y \supset u_{1} \leq z \wedge u_{2} \leq z)[w].\\ \end{array} $$

Now we show that the correspondence is indeed correct. First, let $\mathfrak {F} \vDash \forall y, z, u_{1},$ *u*_2_(*R* *x* *y* *z* ∧ *R* *u*_1_ *u*_2_ *y* ⊃ *u*_1_ ≤ *z* ∧ *u*_2_ ≤ *z*)[*w*]. Suppose *M* is an arbitrary model based on $\mathfrak {F}$, then if *R* *w* *y* *z* ∃*u*_1_ *u*_2_(*R* *u*_1_ *u*_2_ *y* ∧ *P* *u*_1_ ∧ *Q* *u*_2_) both hold, *u*_1_ ≤ *z* and *u*_2_ ≤ *z*, and, by the Hereditary Lemma, *P* *z* and *Q* *z* as desired. Hence, $\mathfrak {F}, w \Vdash p \circ q \rightarrow p \wedge q$. On the other hand, suppose $\mathfrak {F} \nvDash \forall y, z, u_{1}, u_{2} (Rxyz \wedge Ru_{1}u_{2}y \supset u_{1} \leq z \wedge u_{2} \leq z)[w]$. A valuation *V* on $\mathfrak {F}$ such that *V* (*p*) = {*x* : *u*_1_ ≤ *x*} and *V* (*q*) = {*x* : *u*_2_ ≤ *x*} suffices to guarantee that $\mathfrak {F}, w \nVdash p \circ q \rightarrow p \wedge q$ (for $\langle \mathfrak {F}, V \rangle , y \Vdash p \circ q$ but $\langle \mathfrak {F}, V \rangle , z \nVdash p \wedge q$). Observe that in fact the condition simplifies to ∀*y*,*z*(*R* *x* *y* *z* ⊃ *x* ≤ *z* ∧ *y* ≤ *z*) by some manipulations.

### *Example 15*

By the Sahlqvist-van Benthem algorithm,

$$\begin{array}{llll} \mathfrak{F}, w \!\Vdash\! (\textbf{t} \!\rightarrow\! p) \!\rightarrow\! p & \text{iff} & \mathfrak{F} \vDash \forall P \forall y, z (Up_{\leq}(P) \wedge Rxyz\\ && \wedge \forall u, v (Ryuv \wedge \exists b(Ob \wedge b\leq u) \supset Pv) \supset Pz)[w]\\ &\text{iff} & \mathfrak{F} \vDash \forall y, z (Rxyz \wedge \forall u, v (Ryuv \wedge \exists b(Ob \wedge b\leq u) \supset \lambda u_{1}\\ &&. (\exists u_{2}u_{3} (Ryu_{2}u_{3} \wedge \exists b(Ob \wedge b\leq u_{2}) \wedge u_{3} \leq u_{1}))v) \supset \lambda u_{1}\\ &&. (\exists u_{2}u_{3} (Ryu_{2}u_{3} \wedge \exists b(Ob \wedge b\leq u_{2}) \wedge u_{3} \leq u_{1}))z)[w]\\ &\text{iff} & \mathfrak{F} \vDash \forall y, z (Rxyz \wedge \forall u, v (Ryuv \wedge \exists b(Ob \wedge b\leq u) \supset \exists u_{2}u_{3} (Ryu_{2}u_{3} \\ && \wedge \exists b(Ob \wedge b\leq u_{2}) \wedge u_{3} \leq v)) \supset \exists u_{2}u_{3} (Ryu_{2}u_{3} \wedge \exists b(Ob \wedge b\leq u_{2})\\ & & \wedge u_{3} \leq z))[w]\\ & \text{iff} & \mathfrak{F} \vDash \forall y, z (Rxyz \supset \exists u_{2}u_{3} (Ryu_{2}u_{3} \wedge \exists b(Ob \wedge b\leq u_{2}) \!\wedge u_{3} \leq z))[w].\\ \end{array} $$

The latter condition can be written as ∀*x*,*y*,*z*(*O* *x* ∧ *R* *x* *y* *z* ⊃∃*u*_2_ *u*_3_(*R* *y* *u*_2_ *u*_3_ ∧∃*b*(*O* *b* ∧ *b* ≤ *u*_2_) ∧ *u*_3_ ≤ *z*)) when we consider correspondence with respect to the worlds in *O* of a given frame. This condition is actually equivalent to the condition ∀*x*∃*b*(*O* *b* ∧ *R* *x* *b* *x*) corresponding in [4] to (**t** → *p*) → *p* ([4], p. 80, q6).

All that is left is to verify that $\mathfrak {F} \vDash \forall y, z (Rxyz \supset \exists u_{2}u_{3} (Ryu_{2}u_{3} \wedge \exists b(Ob \wedge b\leq u_{2}) \wedge u_{3} \leq z))$ does locally correspond to (**t** → *p*) → *p*. Take a frame $\mathfrak {F}$ such that $\mathfrak {F} \vDash \forall y, z (Rxyz \supset \exists u_{2}u_{3} (Ryu_{2}u_{3} \wedge \exists b(Ob \wedge b\leq u_{2}) \wedge u_{3} \leq z))[w]$. Let *M* be any Routley-Meyer model based on $\mathfrak {F}$. Consider arbitrary worlds *w*_1_,*w*_2_ such that *R* *w* *w*_1_ *w*_2_ holds in *M* and suppose that $M, w_{1} \Vdash \textbf {t} \rightarrow p$, which means that ∀*u*,*v*(*R* *y* *u* *v* ∧∃*b*(*O* *b* ∧ *b* ≤ *u*) ⊃ *P* *v*) holds in *M*. Since *R* *w* *w*_1_ *w*_2_, we can conclude that ∃*u*_2_ *u*_3_(*R* *w*_1_ *u*_2_ *u*_3_ ∧∃*b*(*O* *b* ∧ *b* ≤ *u*_2_) ∧ *u*_3_ ≤ *w*_2_), but then *P* *u*_3_, and, by the Hereditary Lemma, *P* *w*_2_ as desired. On the other hand, suppose that $\mathfrak {F} \nvDash \forall y, z (Rxyz \supset \exists u_{2}u_{3} (Ryu_{2}u_{3} \wedge \exists b(Ob \wedge b\leq u_{2}) \wedge u_{3} \leq z))[w]$, so there are worlds *w*_1_,*w*_2_ such that *R* *w* *w*_1_ *w*_2_ and $\forall u_{2}u_{3} (Rw_{1}u_{2}u_{3} \wedge \exists b(Ob \wedge b\leq u_{2}) \supset u_{3} \nleq w_{2})$. Take any model *M* based on $\mathfrak {F}$ such that $V(p) = \{x \in W : x \nleq w_{2}\}$. *V* (*p*) is upward closed under ≤ thanks to the transitivity of ≤. Also, using the reflexivity of ≤, it is easy to see that $M, w \nVdash (\textbf {t} \rightarrow p) \rightarrow p$, as desired.

The procedure can be used to obtain in a systematic way the following well-known correspondences for the fusion version of some important principles of relevant logic (cf. [14], Table 2.2):

$$\begin{array}{llll} a \leq b \supset \exists y, z (Ryzb \wedge a \leq y \wedge a\leq z) & & xp \rightarrow p \circ p \,\,\, \\ (\text{which is equivalent to } \forall x Rxxx)& & (\text{ Weak Contraction or } \textsf{WL})\\ \forall y, z (a \leq b \wedge Ryza \supset \exists v_{0}, v_{1}, v_{2}, v_{3} (Rv_{0}v_{1}b \wedge & & p \circ q \rightarrow p \circ (p \circ q) \, \\ y \leq v_{0} \wedge Rv_{2}v_{3}v_{1} \wedge y \leq v_{2} \wedge z\leq v_{3})) && (\text{Strong Contraction or} \textsf{W}) \\ (\text{which is equivalent to } \forall x, y, z (Rxyz \supset \exists v (Rxvz \wedge Rxyv)))& & \\ \forall y, z (a \leq b \wedge Ryza \supset z \leq b) & & p \circ q \rightarrow q \,\,\, \\ (\text{which is equivalent to } \forall x, y, z (Rxyz \supset y\leq z))&& (\text{Commuted Weakening or } \textsf{K}^{\prime})\\ \forall y, z (a \leq b \wedge Ryza \supset y \leq b) & & p \circ q \rightarrow p \,\,\, \\ (\text{which is equivalent to } \forall x, y, z (Rxyz \supset x \leq z))&& (\text{Weakening or } \textsf{K}) \\ \forall y, z (a \leq b \wedge Ryza \supset & & p \circ q \rightarrow q \circ p \,\,\, \\ \exists v_{0}, v_{1} (Rv_{0}v_{1}b \wedge z \leq v_{0} \wedge y \leq v_{1})) && (\text{Weak Commutativity or } \textsf{CL}) \\ (\text{which is equivalent to } \forall x, y, z (Rxyz \supset Ryxz)) && \\ \forall y, z, u, v (a \leq b \wedge Ryza \wedge Ruvy \supset & & (p \circ q) \circ r \rightarrow p \circ (q \circ r)\\ \exists v_{0}, v_{1}, v_{2}, v_{3} (Rv_{0}v_{1}b \wedge u \leq v_{0} \wedge Rv_{2}v_{3}v_{1} \wedge v\leq v_{2} \wedge z\leq v_{3})) && (\text{Twisted Associativity or } \textsf{B}^{\prime}) \\ (\text{which is equivalent to } \forall x, y, z, v (\exists w (Rxyw \wedge Rwzv) \supset&& \\ \exists w (Rxwv \wedge Ryzw))x) && \\ \end{array} $$

### **Definition 4**

A formula *ϕ* → *ψ* is called a dual relevant Sahlqvist implication if *ψ* is negative while *ϕ* is a formula built up from negated propositional atoms (i.e. formulas of the form ∼ *p*), triple negated atoms (i.e., formulas of the form ~ *p*), positive formulas, the constant ∼**t** and implications of the form *p* →**t** (for any propositional variable p) using only the connectives ∨,∧ and ∘.

### **Lemma 16**

*Every dual relevant Sahlqvist implication has a local first order* *correspondent on Routley-Meyer frames.*

### *Proof*

We argue essentially as in the proof of Lemma 11. Hence, our purpose is to eliminate all second order quantifiers in a formula of the form:

- (1) ∀*P*_1_,…,*P*_*n*_∀*x*_1_,…,*x*_*k*_(*U* *p*_≤_(*P*_1_)∧…∧*U* *p*_≤_(*P*_*n*_)∧REL∧(T)NAT∧IMP∧∧POS ⊃NEG),

where REL is a conjunction of literals involving only the non-logical symbols R and O, (T)NAT is a conjunction of translations of negated atomic relevant formulas and triple negated atomic relevant formulas, IMP is a conjunction of translations of formulas of the form *p* →**t**, POS is a conjunction of positive formulas and NEG, a negative formula.

As before, we may assume that any unary predicate (corresponding to a propositional variable) appearing in NEG, appears also in the antecedent of (1). Otherwise, instantiate the given unary predicate *P*_*i*_ appearing in NEG (and not in the antecedent of (1)) to *λ* *u*.(*u* ≤ *u*) getting a formula (1) ^′^ −and note that *U* *p*_≤_(*λ* *u*.(*u* ≤ *u*)). As before, using (the contrapositive of) Lemma 3 and the fact that a negative formula is just the (boolean) negation of a positive formula, we see that (1) ^′^ implies (1), so they are indeed equivalent.

We also see that (1) is in fact equivalent to

- (1) ∀*P*_1_,…,*P*_*n*_∀*x*_1_,…,*x*_*k*_(*U* *p*_≤_(*P*_1_)∧…∧*U* *p*_≤_(*P*_*n*_)∧REL∧(T)NAT∧IMP ⊃¬POS∨NEG),

where ¬POS ∨NEG is, of course, a negative formula.

Finally, let *π*_1_(*x*_*i*1_),…,*π*_*k*_(*x*_*ik*_) be all the conjuncts of (T)NAT and IMP in the antecedent of (3) in which the unary predicate *P*_*i*_ occurs. Then if *π*_*j*_(*x*_*ij*_) appears in one of the conjuncts in IMP, it must be a formula of the form ∀*y* *z*(*R* *x*_*ij*_ *y* *z* ∧ *P*_*i*_ *y* ⊃∃*b*(*O* *b* ∧ *b* ≤ *z*)), in which case we define *σ*(*π*_*j*_(*x*_*ij*_)) = *λ* *u*.(∀*z*(*R* *x*_*ij*_ *u* *z* ⊃∃*b*(*O* *b* ∧ *b* ≤ *z*))). On the other hand if *π*_*j*_(*x*_*ij*_) appears is one of the conjuncts in (T)NAT we put $\sigma (\pi _{j}(x_{ij})) = \lambda u. (u \nleq x_{ij}^{*} )$ in case *π*_*j*_(*x*_*ij*_) = ¬*P*_*i*_ *x* *i* *j*∗ and $\sigma (\pi _{j}(x_{ij})) = \lambda u. (u \nleq x_{ij}^{***} )$ in case *π*_*j*_(*x*_*ij*_) = ¬¬¬*P*_*i*_ *x* *i* *j*∗∗∗. Note that *σ* is a well-defined function and that for any *π*_*j*_(*x*_*ij*_), if *π*_*j*_(*x*_*ij*_)[*w*] then, then ∀*y*(*P*_*i*_ *y* ⊃ *σ*(*π*_*j*_(*x*_*ij*_))(*y*))[*w*]. Next define *δ*(*P*_*i*_) = *λ* *u*.(*σ*(*π*_1_(*x*_*i*1_))(*u*) ∧… ∧ *σ*(*π*_*k*_(*x*_*ik*_))(*u*)). Now, if (T)NAT[*w* *w*_1_…*w*_*k*_] and IMP[*w* *w*_1_…*w*_*k*_] then ∀*u*(*P*_*i*_ *u* ⊃ *δ*(*P*_*i*_)(*u*))[*w* *w*_1_…*w*_*k*_]. The remainder of the proof is as before but using again the contrapositive formulation of Lemma 3 and noting that the intersection of a collection of upward closed sets under ≤ is also upward closed under ≤. □

### *Example 17*

Consider the dual relevant Sahlqvist implication (*p* →**t**)∧∼**t** →∼ *p*. Using our Sahlqvist-van Benthem algorithm, we obtain that the following equivalences hold:

$$\begin{array}{llll} \mathfrak{F}, w \!\Vdash\! (p \!\rightarrow\! \textbf{t}) \wedge \sim \textbf{t} \!\rightarrow \sim p & \text{iff} & \mathfrak{F} \vDash \forall P \forall y, z (Up_{\leq}(P) \wedge \forall u, v (Ryuv \wedge Pu \supset \exists b\\ &&(Ob \wedge b\leq v)) \wedge Rxyz \wedge \neg \exists b(Ob \wedge b\leq y^{*}) \supset \neg Pz^{*})[w]\\ & \text{iff} & \mathfrak{F} \vDash \forall y, z (\forall u, v (Ryuv \wedge\\ & & \lambda u_{1}. (\forall u_{2} (Ryu_{1}u_{2} \supset \exists b(Ob \wedge b\leq u_{2}) ))(u) \supset\\ & & \exists b(Ob \wedge b\leq v)) \wedge Rxyz \wedge \neg \exists b(Ob \wedge b\leq y^{*}) \supset\\ && \neg \lambda u_{1}. (\forall u_{2} (Ryu_{1}u_{2} \supset \exists b(Ob \wedge b\leq u_{2}) ))(z^{*}))[w]\\ & \text{iff} & \mathfrak{F} \vDash \forall y, z (\forall u, v (Ryuv \wedge \forall u_{2} (Ryuu_{2} \supset \exists b(Ob \wedge b\leq u_{2}) )\\ &&\supset \exists b(Ob \wedge b\leq v) \wedge Rxyz \wedge \neg \exists b(Ob \wedge b\leq y^{*}) \supset \\&&\neg \forall u_{2} (Ryz^{*}u_{2} \supset \exists b(Ob \wedge b\leq u_{2})))[w]\\ & \text{iff} & \mathfrak{F} \vDash \forall y, z (Rxyz \wedge \neg \exists b(Ob \wedge b\leq y^{*}) \supset \\&& \neg \forall u_{2} (Ryz^{*}u_{2} \supset \exists b(Ob \wedge b\leq u_{2}) ))[w] \\ &\text{iff} & \mathfrak{F} \vDash \forall y, z (Rxyz \wedge \neg \exists b(Ob \wedge b\leq y^{*}) \\&&\supset \exists u_{2} (Ryz^{*}u_{2} \wedge \neg \exists b(Ob \wedge b\leq u_{2})))[w].\\ \end{array} $$

We now show that the above correspondence is indeed correct. First let $\mathfrak {F} \vDash \forall y, z (Rxyz \wedge \neg \exists b(Ob \wedge b\leq y^{*}) \supset \exists u_{2} (Ryz^{*}u_{2} \wedge \neg \exists b(Ob \wedge b\leq u_{2})))[w]$. Then if *U* *p*_≤_(*P*), *R* *w* *y* *z*, ∀*u*,*v*(*R* *y* *u* *v* ∧ *P* *u* ⊃∃*b*(*O* *b* ∧ *b* ≤ *v*)) and ¬∃*b*(*O* *b* ∧ *b* ≤ *y* ^∗^), it must be that ∃*u*_2_(*R* *y* *z* ^∗^ *u*_2_ ∧¬∃*b*(*O* *b* ∧ *b* ≤ *u*_2_)). Thus if *P* *z* ^∗^, ∃*b*(*O* *b* ∧ *b* ≤ *u*_2_), which is a contradiction, so ¬*P* *z* ^∗^. Consequently $\mathfrak {F}, w \vDash (p \rightarrow \textbf {t}) \wedge \sim \textbf {t} \rightarrow \sim p$, as desired. On the other hand suppose that $\mathfrak {F} \nvDash \forall y, z (Rxyz \wedge \neg \exists b(Ob \wedge b\leq y^{*}) \supset \exists u_{2} (Ryz^{*}u_{2} \wedge \neg \exists b(Ob \wedge b\leq u_{2})))[w]$, so $\mathfrak {F} \vDash \exists y, z (Rxyz \wedge \neg \exists b(Ob \wedge b\leq y^{*}) \wedge \forall u_{2} (Ryz^{*}u_{2} \supset \exists b(Ob \wedge b\leq u_{2})))[w]$. Take any model *M* based on $\mathfrak {F}$ with a valuation such that *V* (*p*) = {*x* : *z* ^∗^≤ *x*}. Now, if *R* *y* *u*_1_ *u*_2_ and *u*_1_ ∈ *V* (*p*), that is, *z* ^∗^≤ *u*_1_, we that, by p3, *R* *y* *z* ^∗^ *u*_2_, and hence, ∃*b*(*O* *b* ∧ *b* ≤ *u*_2_). This shows that $M, y \Vdash (p \rightarrow \textbf {t}) \wedge \sim \textbf {t}$ while $M, z \nVdash \sim p$, which implies that $\mathfrak {F}, w \nvDash (p \rightarrow \textbf {t}) \wedge \sim \textbf {t} \rightarrow \sim p$.

### **Definition 5**

A relevant Sahlqvist formula is any formula built up from (dual) relevant Sahlqvist implications, propositional variables, and negated propositional variables using ∧, the operations on formulas Θ (for any propositional variable free relevant formula *𝜃*) defined by Θ(*ϕ*) = *𝜃* → *ϕ*, and applications of ∨ where the disjuncts share no propositional variable in common.

### **Theorem 18**

*Every relevant Sahlqvist formula has a local first order correspondent on* *Routley-Meyer frames.*

### *Proof*

Immediate from Lemma 11, Lemma 16 and Lemma 9. The only thing the reader should note is that $\mathfrak {F}, w \Vdash p$ iff $\mathfrak {F} \vDash x \nleq x[w]$ and $\mathfrak {F}, w \Vdash \sim p$ iff $\mathfrak {F} \vDash x^{*} \nleq x^{*} [w]$. □

## Conclusion

In this paper we have defined a fragment of relevant languages analogue to the Sahlqvist fragment of modal logic. We then went to establish that every class of Routley-Meyer frames definable by a formula in this fragment is actually elementary. This isolates a modest but remarkable collection of relevant formulas. We also showed that there are properties of Routley-Meyer frames definable by relevant formulas which are not first order axiomatizable.

## Footnotes

1 The Routley-Meyer framework remains today the most prominent non-algebraic approach to the semantics of propositional relevant languages (cf. [4, 8]). Recent applications (e.g., [15, 19]) are easy to find. The Routley-Meyer semantics has also been given an appropriate philosophical justification in [2] and much more recently in [9]. If anything comes close to being the “standard” relational semantics for propositional relevant logics, it surely is the Routley-Meyer approach.

2 Many thanks to an anonymous referee who pointed this out.

3 Note that in [7] elementary classes are called “basic elementary”.
